# cAMP Signalling Pathway in Biocontrol Fungi

**DOI:** 10.3390/cimb44060179

**Published:** 2022-06-04

**Authors:** Zhan-Bin Sun, Shu-Fan Yu, Chu-Lun Wang, Ling Wang

**Affiliations:** 1 School of Light Industry, Beijing Technology and Business University, Beijing 100048, China; ysf18437906517@163.com (S.-F.Y.); wcl20010424@163.com (C.-L.W.); 2 Key Laboratory of Integrated Pest Management on Crops in Central China, Ministry of Agriculture and Rural Affairs, Hubei Key Laboratory of Crop Disease, Insect Pests and Weeds Control, Institute of Plant Protection and Soil Fertilizer, Hubei Academy of Agricultural Sciences, Wuhan 430064, China

**Keywords:** cAMP signalling pathway, biocontrol, G-protein system, adenylate cyclase, cAMP-dependent protein kinase, transcription factor

## Abstract

Biocontrol is a complex process, in which a variety of physiological and biochemical characteristics are altered. The cAMP signalling pathway is an important signal transduction pathway in biocontrol fungi and consists of several key components. The G-protein system contains G-protein coupled receptors (GPCRs), heterotrimeric G-proteins, adenylate cyclase (AC), cAMP-dependent protein kinase (PKA), and downstream transcription factors (TFs). The cAMP signalling pathway can regulate fungal growth, development, differentiation, sporulation, morphology, secondary metabolite production, environmental stress tolerance, and the biocontrol of pathogens. However, few reviews of the cAMP signalling pathway in comprehensive biocontrol processes have been reported. This work reviews and discusses the functions and applications of genes encoding each component in the cAMP signalling pathway from biocontrol fungi, including the G-protein system components, AC, PKA, and TFs, in biocontrol behaviour. Finally, future suggestions are provided for constructing a complete cAMP signalling pathway in biocontrol fungi containing all the components and downstream effectors involved in biocontrol behavior. This review provides useful information for the understanding the biocontrol mechanism of biocontrol fungi by utilising the cAMP signalling pathway.

## 1. Introduction

Biological control is a complex process, including the recognition of pathogens by biocontrol agents, biocontrol-related signal transduction by different cell signal transduction pathways, and finally, biocontrol effects through the relevant response of biocontrol-related signals by biocontrol agents, including the secretion of cell wall degradation enzymes to destroy the cell walls of pathogens or the production of antibiotics and toxins to inhibit or kill pathogens [1,2,3,4]. Among these processes, signal transduction plays crucial roles in biocontrol. Signal transduction pathways in cells mainly include the heterotrimeric G protein signalling pathway, mitogen-activated protein kinase (MAPK) signalling pathway, and cAMP signalling pathway [1,5,6]. The G protein and MAPK signalling pathways in biocontrol agents have been well reviewed; however, seldom reviews of the cAMP signalling pathway in comprehensive biocontrol agents have been reported.

**Figure 1:**
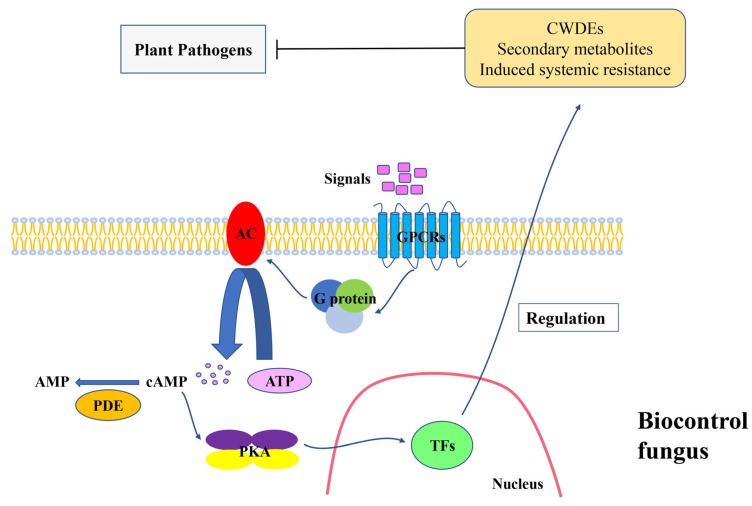
cAMP signalling pathway in biological control. GRCRs: G-protein coupled receptors; AC: Adenylate cyclase; PDE: Phosphodiesterase; PKA: cAMP-dependent protein kinase A; TFs: Transcription factors; CWDES: Cell-wall-degrading enzymes. Pathogen-related signals are transmitted into cells through GPCRs, activate G-proteins, and then stimulate AC. The activated AC converts ATP to cAMP, then, cAMP stimulates PKA, and finally, the activated PKA regulates the expression activities of downstream proteins, such as transcription factors, resulting in the biocontrol of pathogens.

Cyclic adenosine 3′5′ monophosphate (cAMP) is a second messenger in both prokaryotes and eukaryotes and is involved in a variety of biological processes [7]. The activity of cAMP is regulated by two enzymes, adenylate cyclase (AC) and phosphodiesterase (PDE). AC catalyses ATP-synthesised cAMP, while PDE degrades cAMP [8]. The cAMP signalling pathway consists of several key components: the G-protein system contains G-protein coupled receptors (GPCRs) and heterotrimeric G-proteins, AC and cAMP-dependent protein kinase (PKA), and downstream effectors such as transcription factors [9]. Extracellular signal transduction undergoes the following processes in the cAMP signalling pathway: extracellular signals are transmitted into cells through GPCRs, and activated heterotrimeric G-proteins stimulate AC, leading to the production of cAMP. Then, PKA is triggered by cAMP stimulation, and finally, the activated PKA regulates the expression activities by phosphorylation of downstream proteins, such as transcription factors, resulting in the modulation of multiple biological processes [10] (Figure 1).

The cAMP signalling pathway has been widely reported in medicine. The cAMP signalling pathway is involved in neurobiology, affecting astrocyte morphology, regulating learning ability, memory formation, numerous neuronal functions, and ultimately behaviours, and participating in psychiatric and neurodegenerative illness [11,12,13,14,15,16,17]. The cAMP signalling pathway is critical in adjusting the development of cardiac fibrosis and the functions of cardiac fibroblasts and is closely related to the contraction rate and force of the heart [18,19,20,21,22]. Moreover, the cAMP signalling pathway could influence hypertrophy and hyperplasia of the pituitary gland and insulin resistance in obesity [23,24,25,26,27]. Therefore, the cAMP signalling pathway is generally used as a therapeutic target for the treatment of numerous illnesses, such as alcohol-associated liver disease [28,29,30,31,32,33].

In addition to its crucial roles in human health and medicine, the cAMP signalling pathway can regulate fungal growth, differentiation, development, conidiation, biofilm formation, sexual mating, and white-opaque switching in some specific fungi [34,35,36,37,38]. The cAMP signalling pathway is also closely related to the virulence of fungal plant pathogens, animal pathogens and malaria parasites [39,40,41,42,43]. Additionally, the cAMP signalling pathway plays important roles in biocontrol agents, such as *Trichoderma* spp., *Metarhizium anisopliae*, and *Beauveria bassiana*, by influencing the formation of appressoria or secretion of cell-wall-degrading enzymes and secondary metabolites [44,45,46,47].

In this review, the roles and applications of each component in the cAMP signalling pathway of biocontrol fungi are interpreted and discussed in detail. The review provides useful insights for further application of biocontrol by utilisation of the cAMP signalling pathway.

## 2. G Protein System

The G protein system is an upstream element of the cAMP signalling pathway. The G protein system mainly contains two components, GPCRs and heterotrimeric G protein. GPCRs contain seven transmembrane domains, which can recognise external signals [48]. Heterotrimeric G protein is highly conserved among organisms and is composed of α, β, and γ subunits [49,50]. Among the three components, the G protein α subunit (Gα) is closely involved in the cAMP signalling pathway. Three subgroups exist in Gα, and subgroups I and III can inhibit and stimulate AC, respectively [51,52]. Extracellular signals are recognised by GPCRs, and ligand-binding GPCRs exchange GDP–GTP on Gα and release the G protein βγ complex. Then, the activated Gα stimulates AC and catalyses ATP synthesis of cAMP [53]. G proteins are critical for morphogenesis, growth, development, mating, virulence, and secondary metabolism [54,55,56,57].

The G protein system was also shown to be involved in the biocontrol ability of a number of fungi, such as *Trichoderma* spp., *Clonostachys* spp., *Coniothyrium* spp., *Metarhizium* spp., and *Beauveria* spp. (Table 1). *Trichoderma* spp., *Clonostachys* spp., and *Coniothyrium* spp. are typical mycoparasitism biocontrol agents, with the main targets being pathogenic fungi. *Metarhizium* spp. and *Beauveria* spp. are commonly known as entomopathogenic biocontrol agents, with the main targets being pathogenic insects. Among these biocontrol agents, *Trichoderma* is the most widely studied in terms of the function of the G protein system. The absence of some GPCR genes reduced the biocontrol ability of *Trichoderma* against pathogens. Silencing of the seven-transmembrane GPCR-encoding gene *gpr1* in *T. atroviride* resulted in the mutant losing the ability to attack and parasite the pathogens *Rhizoctonia solani*, *Botrytis cinerea,* and *Sclerotium sclerotiorum*. A study found that the quantity of some important antifungal metabolites, such as 6-pentyl-α-pyrone, was significantly reduced in the mutant, and the expression levels of some cell-wall-degrading enzyme encoding genes that are very important for *T. atroviride* mycoparasitism were also dramatically altered in the mutant [58].

Gα can directly activate AC to synthesise cAMP. Gα-encoding genes in different *Trichoderma* species, which exhibits mycoparasitism ability. Reithner et al. [59] deleted the Gα subunit-encoding gene *tga1* in *T. atroviride* and found that the mycoparasitic abilities of the mutant against the pathogens *R. solani*, *B. cinerea,* and *S. sclerotiorum* were lost, and the chitinase activities and 6-pentyl-α-pyrone production capacity were reduced when compared with those of the wild-type strain. A mutant of *T. reesei* carried a constitutively activated version of *gna3*, which encodes for the Gα subunit, and exhibited an improved antagonistic ability to *Pythium ultimum* [60]. Overexpression of *tga1* in *T. atroviride* could enhance the mycoparasitism capacity of the transformant to *R. solani* [61]. Similar results were found with the disruption of another Gα subunit-encoding gene, *tga3*, in *T. atroviride*, and the mycoparasitic abilities of the mutant against *R. solani* and *B. cinerea* were lost, Moreover, the chitinase activity in the mutant was reduced, and the infection structures in the mutant were not formed [62]. The biocontrol roles of Gα-encoding genes in other *Trichoderma* species have also been clarified. The absence of the Gα-encoding gene *tgaA* in *T. virens* resulted in the mutant having a reduced ability to antagonise *S. rolfsii* and losing the ability to parasitise the sclerotia of *S. rolfsii* [63]. In *T. harzianum*, the deletion of two Gα subunit-encoding genes, *thga1* and *thga3*, resulted in significantly altered mycoparasitic abilities of ∆thga1 and ∆thga3 against *R. solani*, and chitinase activity was markedly reduced in ∆thga3 [64,65].

In addition to mycoparasitism agents, the role of the G protein system in entomopathogenic fungal agents has also been deeply studied. GCPRs and G protein are vital for the virulence of entomopathogenic fungal agents. Shang et al. [66] deleted ten GPCR-encoding genes in *M. robertsii* and found that the mutant ∆MrGpr8 lost the ability to form appressoria and infect insects. Disruption of the Gα subunit-encoding gene *MrGPA1* in *M. robertsii* could affect the formation of appressoria and the expression level of cuticle-penetration-related genes and reduce the virulence of the mutant to *Galleria mellonella* larvae [67]. In *B. bassiana*, GPCRs also play crucial roles in virulence to insects. Knockout of *BbGPCR3*, which encodes a GPCR in *B. bassiana*, could lead to a reduced virulence in topical insect assays [68].

Moreover, the expression levels of some important G protein system genes are differentially expressed during the biological control process. GCPR- and Gα-encoding genes were upregulated in *C. rosea* controlling *Helminthosporium solani* [69]. The gene encoding the PTH11-like GPCR was preferentially expressed in *C. minitans* when parasitising sclerotia of *S. sclerotiorum* [70]. The Pth11-like GPCR-encoding gene was upregulated in *B. bassiana* infecting *Anopheles stephensi* adult mosquitoes [71].

**Table 1:** Biocontrol related genes involved in the cAMP signalling pathway of biocontrol fungi.

Protein		Gene	Biocontrol Fungi	Pathogens
**G protein system**	G-protein alpha subunit	*tgaA*	*Trichoderma virens*	*Sclerotium rolfsii* [63]
	G-protein alpha subunit	*thga1*	*T. harzianum*	*Rhizoctonia solani* [65]
	G-protein alpha subunit	*thga3*	*T. harzianum*	*R. solani* [64]
	G-protein alpha subunit	*tga3*	*T. atroviride*	*Botrytis cinerea*, *R. solani* [62]
	G-protein alpha subunit	*tga1*	*T. atroviride*	*R. solani*, *B. cinerea*, *S. sclerotiorum* [59]
	Seven-transmembrane receptor Gpr1	*gpr1*	*T. atroviride*	*R. solani*, *B. cinerea*, *S. sclerotiorum* [58]
	G-protein alpha subunit	*gna3*	*T. reesei*	*Pythium ultimum* [60]
	G-protein alpha subunit	*MrGpa1*	*Metarhizium robertsii*	*Galleria mellonella* [67]
	G-protein coupled receptor	*MrGpr8*	*M. robertsii*	*G. mellonella*, *Tenebrio molitor, Bombyx mori* [66]
	G-protein coupled receptor	*BbGPCR3*	*Beauveria bassiana*	*G. mellonella* [68]
	G-protein coupled receptor	BBA_00828 BBA_05185	*B. bassiana*	*Anophele stephensi* [71]
	G-protein coupled receptor	CmEST-463	*Coniothyrium minitans*	*S. sclerotiorum* [70]
	G-protein coupled receptor	BN869_T00001016_1	*Clonostachys rosea*	*Helminthosporium solani* [69]
**Adenylate cyclase**	Adenylate cyclase	*tac1*	*T. virens*	*S. rolfsii*, *R. solani*, *Pythium* sp. [72]
	Adenylate cyclase	*MaAC*	*M. acridum*	*Locusta migratoria* [73]
	Adenylate cyclase	*MrAC*	*M. robertsii*	*T. molitor* [74]
	Adenylate cyclase	*BcAC*	*B. bassiana*	*G. mellonella* [74]
**Phosphodiesterase**	Phosphodiesterase	*AopdeH*	*Arthrobotrys oligospora*	*Caenorhabditis elegans* [75]
**Protein kinase A**	Protein kinase A	*MaPKA1*	*M. anisopliae*	*G. mellonella* [76]
	Protein kinase A	CMZSB_03553	*C. minitans*	*S. sclerotiorum* [77]
**Transcription factor**	Transcription factor	*MrStuA*	*M. rileyi*	*Spodoptera litura* [78]
	Transcription factor	*MaSom1*	*M. acridum*	*L. migratoria manilensis* [79]

## 3. Adenylate Cyclases

Adenylate cyclases (ACs) are highly conserved among organisms. In vertebrates, ACs commonly contain nine transmembrane and one soluble isoforms [33,80]. Several functional domains, including leucine-rich repeat domains, cyclase catalytic domains, and Ras-association domains, exist in ACs [81,82,83]. AC plays a crucial role in the cAMP signalling pathway. AC is activated by Gα and then catalyses ATP conversion to cAMP. AC can regulate the growth, development, mating, morphological characteristics, conidiation, metabolite production, and resistance to environmental stress of fungi [84,85,86,87], as well as the pathogenicity of fungal pathogens, including the formation of appressoria and virulence to the host [88,89,90]. Moreover, in the medical area, ACs can regulate pathological cardiac fibrosis and monocyte inflammation [91,92,93].

Adenylate cyclases have been reported to be involved in the biocontrol ability of mycoparasitic fungi, such as *Trichoderma* spp. (Table 1). Deletion of the adenylate cyclase-encoding gene *tac1* in *T. virens* reduced the cAMP level. The absence of *tac1* influenced the growth rate, morphology, sporulation, conidial germination, and secondary metabolite production ability of *T. virens*, as well as its confrontation ability against *S. rolfsii*, *R. solani,* and *Pythium sp*. [72]. The cAMP level and coil number of *T. harzianum* were dramatically increased when *T. harzianum* was in close contact with *R. solani* [94]. The deletion of G protein-encoding genes in *T. atroviride* or *T. harzianum* could decrease the cAMP levels and led to a weak mycoparasitic ability of *T. atroviride* against *R. solani*, *B. cinerea*, *S. sclerotiorum,* and *T. harzianum* against *R. solani when* compared with the wild strains [62,65].

In entomopathogenic fungi, ACs can influence the biocontrol ability of insects. Silencing the AC-encoding gene *MaAC* in *M. acridum* reduced cAMP levels and influenced growth and tolerance to environmental stresses, including heat shock, UV-B radiation, and oxidative and osmotic stress, in addition to reducing virulence in *Locusta migratoria* adults [73]. Deletion of the AC-encoding genes *BcAC* in *B. bassiana* and *MrAC* in *M. robertsii* affected the conidiation and response to multiple environmental stresses of the two strains and influenced the biocontrol ability of *M. robertsii* to *Tenebrio molitor* third-instar larvae and *B. bassiana* to *Galleria mellonella* larvae [74].

Phosphodiesterase (PDE) could catalyse the hydrolysis of cAMP [18]. PDEs have been reported to be involved in biocontrol behavior. Ma et al. [75] deleted a PDE-encoding gene *AopdeH* in the nematode-trapping fungus *Arthrobotrys oligospora* and found that the mutant had defect in biocontrol ability against nematodes and stress response, and its morphological characteristics included conidiation, mycelial growth and trap formation.

## 4. cAMP-Dependent Protein Kinase A

The exchange protein activated by cAMP (EPAC) and PKA are effectors of cAMP in mammalian cells [32]. Between the two effectors, PKA has been wildly studied. PKA is a serine/threonine kinase that consists of two regulatory subunits and two catalytic subunits, with the anchoring protein being A-kinase anchoring proteins (AKAPs) [18,95]. All the subunits are conserved among organisms. Without cAMP binding, the regulatory subunits are combined with catalytic subunits and do not have kinase activity. When signals are transduced through the cAMP signalling pathway, cAMP binding leads to conformational changes in PKA. Regulatory subunits of PKA bind to cAMP, and the catalytic subunits are dissociated. The activated catalytic subunits regulate the expression levels of downstream genes through phosphorylation and ultimately regulate biological behaviours [96]. PKA could influence the growth, development, metabolism, and morphological characteristics of microorganisms, as well as the virulence or invasion ability of pathogens to the host [97,98]. However, few studies of the biocontrol functions of PKA have been reported (Table 1).

Disruption of the class I PKA catalytic subunit gene *MaPKA1* in *M. anisopliae* resulted in a series of changes. The growth, tolerance to oxidative stress, conidial adhesion, and appressorium formation ability were influenced in the mutant compared with those in the wild-type strain. Moreover, the virulence of the mutant to *G. mellonella* was significantly reduced. This study found that deletion of *MaPKA1* influenced the expression levels of numerous pathogenicity genes, including sterol synthesis, tetraspanin-like protein, subtilisin-like protease, and squalene epoxydase 1 genes, which are involved in the biocontrol processes of appressorium and penetration peg formation, insect cuticle degradation and resistance to antifungal compounds [76]. In addition, the gene encoding cAMP-dependent protein kinase was dramatically differentially expressed in *C. minitans,* infecting *S. sclerotiorum* at different time points [77].

## 5. Transcription Factors

Transcription factors (TFs) are downstream components of the cAMP signalling pathway. TFs are activated through phosphorylation by catalytic subunits of PKA and combine with the related cis-acting elements to regulate the expression of target genes. To date, 61 TF families have been found in Fungal Transcription Factor Database (http://ftfd.snu.ac.kr/index.php?a=view, accessed on 1 January 2022). The Zn2Cys6 family has the highest numbers of TFs, and the bZIP, C2H2 zinc finger, Forkhead, GATA type zinc finger, heteromeric CCAAT factors, HMG, homeobox, homeodomain-like, Myb, winged helix repressor DNA-binding and zinc finger (CCHC-type) TF families are the most abundant in fungal species. Among the reported fungi, *Fusarium oxysporum* f. sp. lycopersici had the highest number of TFs.

TFs are involved in numerous fungal physiological processes. TFs influence the growth, development, morphological characteristics, production of secondary metabolites, and environmental stress tolerance of fungi [99,100]. Moreover, TFs play crucial roles in biocontrol behavior. Although TFs have been wildly reported to be involved in the biocontrol ability against pathogens in different fungal species, only a few biocontrol-related TF-encoding genes were reported as the downstream effector in the cAMP signalling pathway (Table 1). The APSES-type transcription factor gene StuA homologs targets of the cAMP signalling pathway in fungi. Deletion of *MrStuA* in *M. rileyi* influenced the virulence of the mutant to *Spodoptera litura* larvae [78]. Disruption of a downstream transcriptional factor of cAMP signalling pathway gene *MaSom1* could influence *M. acridum* infection of *L. migratoria manilensis* [79].

Besides the above TF-encoding genes that are clearly involved in the cAMP signalling pathway, there are still many TF-encoding genes that belong to different TF families and exhibit biocontrol activity, and they might participate in cAMP signalling pathway. TFs that belong to the bZIP family have been reported to be activated through the cAMP signalling pathway [101]. The bZIP transcription factor gene *Mrap1* null mutant of *M. rileyi* altered the virulence to *Spodoptera litura* larvae [102]. In *M. robertsii*, the absence of bZIP transcription factor gene *MBZ1* also affected the virulence of *M. robertsii* to silkworm and wax moth larvae [103]. Deletion of the bZIP TF gene *BbHapX* affected the virulence of *B. bassiana* to *G. mellonella* larvae [104]. Moreover, bZIP transcription factor-encoding genes were involved in different mycoparasitism stages of *C. minitans*, and were differentially expressed during the process of *T. koningii* against *S. rolfsii* [105].

Additionally, zinc finger TF-encoding genes were also reported to be involved in the cAMP signalling pathway [106]. Absence of the C2H2 transcription factor gene *Msn2* and GATA-type transcription factor gene *MrNsdD* would affect the virulence of *M. rileyi* to *Spodoptera litura* larvae [107,108]. In *M. robertsii*, disruption of *PacC* homologue transcription factor gene *MrpacC* could influence the virulence of *M. robertsii* to silkworm larvae [109]. In addition, deletion of the PacC gene *MaPacC* could affect *M. acridum* infection of *Locusta migratoria manilensis* [110]. In *B. bassiana*, zinc finger TFs also play vital roles in biocontrol. Deletion of the Zn(II)2Cys6 TF genes *BbTpc1* and *BbThm1*, Far/CTF1-type zinc finger TF genes *Bbctf1*α and *Bbctf1*β in *B. bassiana* would influence the virulence of the mutant to *G. mellonella* larvae [111]. The biocontrol ability of *B. bassiana’s* infection of *Tenebrio molitor* larvae and adults and *G. mellonella* larvae was influenced after the PacC TF gene *pacC* was disrupted [112]. The Zn(2)-C6-type transcription factor-encoding gene was upregulated in *Duddingtonia flagrans,* infecting nematodes at different trapping stages [113]. In *C. minitans*, deletion of the PacC TF gene *CmpacC* reduced the mycoparasitic activity of *C. minitans* against *S. sclerotiorum* and the activities of the cell-wall-degrading enzymes chitinase and β-1,3-glucanase [114]. Similar results have been found in *Trichoderma* and *Clonostachys*. Deletion of a TF gene *pacC* in *T. virens* would affect the overgrowth and parasitism ability of *R. solani* and *S. rolfsii* to pathogens, respectively [115]. Disruption of the TF genes *pacC* in *C. rosea* attenuated their virulence to nematodes [116].

The cAMP signalling pathway could also regulate the expression of heat-shock TF-encoding genes [117]. Heat-shock TFs play important roles in the virulence of *B. bassiana*. Deletion of three heat-shock TFs encoding genes *Hsf1*, *Sfl1*, and *Skn7* in *B. bassiana* could influence the virulence to *G. mellonella* larvae [118]. In *M. robertsii*, the absence of the heat-shock TF-encoding gene *MrSkn7*, would affect the virulence of *M. robertsii* to wax moth larvae [119]. Similar phenomenon was found in *Hirsutella minnesotensis*; the disruption of the heat-shock TF gene *SKN7* could attenuate the virulence of *H. minnesotensis* to nematodes [120]. Moreover, the expression of MADS-box TFs could be regulated by the cAMP signalling pathway [121]. In *B. bassiana*, absence of the MADS-box TF gene *Bbmcm1* could influence the virulence of the mutant to *G. mellonella* larvae [122]. The MADS-box TF gene *Rlm1* was verified to play important roles in the *Candida oleophila* control of *B. cinerea* by gene deletion, and the mutant showed a reduced biocontrol efficacy against postharvest grey mould of kiwifruit [123].

## 6. Conclusions and Perspectives

The cAMP signalling pathway is an important signal transduction pathway. The cAMP signalling pathway can regulate the physiological characteristics of fungi, including growth, development, differentiation, sporulation, morphology, and secondary metabolite production. The cAMP signalling pathway could influence the tolerance of fungi to environmental stress through external signals, affect the pathogenicity of pathogens to the host, or affect the biocontrol ability of biocontrol fungi against different kinds of pathogens. When compared with reviews of other signal transduction pathways, reviews of cAMP signalling pathways in biological control are rare. Therefore, this review focuses on the function of each component of the cAMP signalling pathway in biocontrol fungi in the biocontrol process. This review provides a basis for understanding the mechanism of the cAMP signalling pathway involved in biological control.

Currently, studies of cAMP signalling pathways in biocontrol mainly focus on the function of individual components, such as the G protein system, AC, PKA, and transcription factors in biocontrol. However, the complete cAMP signalling pathway in comprehensive biocontrol fungi has seldom been studied. Because numerous genes encoding cAMP signalling pathway components exist in the genomes of biocontrol fungi, it is critical to understand how all cAMP signalling pathway components are connected. Studies of the cAMP signalling pathway in biocontrol could be conducted with the following steps.

(1) The expression levels of cAMP signalling pathway upstream genes, such as the G protein system components (GPCRs and G protein), should be investigated in biocontrol-fungi-infecting pathogens. The expression levels could be detected through transcriptome sequencing or proteomic analysis or directly monitored by RT-qPCR. Among all the G protein system-encoding genes, those genes with significant differential expression would be selected for gene knockout or silencing to clarify their functions in biocontrol.
(2) The expression levels of all AC-encoding genes should be investigated in the wild-type strain and G protein system null-mutant-infecting pathogens. AC genes that are dramatically differentially expressed after G protein system-encoding gene deletion would be chosen for the gene functional analysis. Gene knockout or silencing was used to investigate the functions of AC-encoding genes in biocontrol. Thus, genes encoding the G protein system and AC in the same cAMP signalling pathway would be connected.
(3) Accordingly, PKA-encoding genes involved in the consistent cAMP signalling pathway would be selected using the above strategy through the process of comparison of the wild strain with the AC null mutant against pathogens. Hence, genes encoding the G protein system, AC, and PKA in the same cAMP signalling pathway would be connected.
(4) The most important components in the cAMP signalling pathway are the downstream TFs. Because a variety of TF families exist, identifying TF-encoding genes consistent with the same cAMP signalling pathway using the above strategy is very important. Thus far, a complete cAMP signalling pathway involved in biocontrol behaviour has been constructed.
(5) Finally, the expression levels of some downstream effector protein genes, such as genes encoding cell-wall-degrading enzymes or secondary metabolite production, should be investigated through comparison of the wild-type strain with the TF null mutant against pathogens. Future work is vital to deeply clear the mechanism of the biocontrol signals transduced through the cAMP signalling pathway in biocontrol fungi.

## Data Availability

Not applicable.
